# Does analgesic overuse matter? Response to OnabotulinumtoxinA in patients with chronic migraine with or without medication overuse

**DOI:** 10.1186/s40064-015-1386-8

**Published:** 2015-10-09

**Authors:** Fayyaz Ahmed, Hassan W. Zafar, Alina Buture, Modar Khalil

**Affiliations:** Department of Neurology, Hull Royal Infirmary, Anlaby Road, Hull, HU3 2JZ UK

## Abstract

Chronic migraine affects 2 % of the population and has substantial impact on quality of life and considerable burden on healthcare resources. 50–80 % patients with chronic migraine have excessive consumption of analgesic medications. Withdrawal of analgesics is often advised before commencing preventive treatments. However, some headache experts recommend preventive treatments alongside analgesic withdrawal. 434 patients with chronic migraine attending the Hull Headache Clinic who received OnabotulinumtoxinA as preventive treatment were stratified to those with or without analgesic overuse. Data was collected through a dedicated headache diary and analysed for headache and migraine days reduction and for an increment in headache-free days in the month post treatment. The data shows no difference in the therapeutic outcome in patients with or without analgesic overuse with substantial reduction in headache and migraine days and an increment in headache-free days in both groups in a real-life clinical setting. OnabotulinumtoxinA is equally effective in patients with chronic migraine with or without analgesic overuse.

## Background

Chronic migraine (CM), defined as headache on ≥15 days/month for ≥3 months of which ≥8 days meet criteria for migraine with or without aura or responds to migraine-specific treatment (IHS 2013). CM is a highly disabling primary headache disorder that affects approximately 2 % of the general population (Natoli et al. 2010). Patients with CM have reduced quality of life (QoL) (Bigal et al. 2008; Lipton et al. 2001); increased risk of anxiety, depression and chronic pain (Victor et al. 2010) and use more healthcare resources than those with episodic migraine (Blumenfield et al. 2011). CM has significant health, social and economic consequences (Munakata et al. 2009). Patients with CM are advised to treat their headache attacks with analgesics and given preventive treatments, taken daily irrespective of whether or not headache is present (Mathew 1993). A significant proportion of patients with CM have a high intake of analgesic medications and around 50–80 % of patients with CM attending specialist headache clinic have analgesic overuse (Deiner and Limmroth 2004; Bigal et al. 2004). Excessive consumption of analgesics may lead to development of medication overuse headache (MOH), although it remains uncertain whether this is a consequence or a cause of CM (Dodick and Freitag 2006; Negro and Martelletti 2011). Many headache experts recommend withdrawal of the overused medication before commencing preventive treatment, although this is not based on randomised, placebo-controlled trials (Hagen et al. 2009) and for many patients in real life clinical practice, this is not a pragmatic solution. It is argued that preventive treatment is only fully effective after stopping the overused medication and maximise response to acute medication (Zeeberg et al. 2006). The issue of high prevalence of analgesic overuse in CM is acknowledged by the International Headache Society and allow their inclusion in clinical trials provided they are stratified accordingly (Silberstein et al. 2008). In real life and in the absence of robust evidence, the choice of preventive treatment before or after withdrawal remains with the treating physician.

OnabotulinumtoxinA remains the only licensed medication for the prevention of CM. Its efficacy and safety has been shown in the phase III Research Evaluating Migraine Prophylaxis Therapy (PREEMPT) clinical programme (Aurora et al. 2011; Dodick et al. 2010; Aurora et al. 2010; Diener et al. 2010; Blumenfield et al. 2010). The sub-group analysis of the PREEMPT data showed this to be equally effective in patients with CM with medication overuse (Silberstein et al. 2013). The Hull prospective data in real-life clinical practice supported PREEMPT findings for the efficacy and safety of OnabotulinumtoxinA as preventive treatment in CM patients (Khalil et al. 2014, 2015). Around 50 % patients in the prospective data had medication overuse. This paper reports the response to OnabotulinumtoxinA in patients with CM with medication overuse in a real-life clinical setting.

## Methods

The data was collected from the Hull Migraine Clinic where patients were treated free of charge on the National Health Service (NHS) following recommendations from the National Institute for Health and Care Excellence (NICE) through their technology appraisal guidance (TAG260) on the use of OnabotulinumtoxinA in adult patients with CM (NICE Technology Appraisal guidance 2012). The Hull Migraine Clinic (Hull Royal Infirmary and Spire Hospital Hull and East Riding) is a tertiary headache centre that sees 1200 new headache referrals each year from across the North of England.

### Study participants

Adult patients with CM defined by the ICHD II criteria (IHS 2004) attending the Hull Migraine Clinic between 1st July 2010 and 31st March 2015 were treated with OnabotulinumtoxinA after discussion of all available options. As per NICE guidelines, all patients had failed to respond to at least three preventive treatments and were treated free at the point of entry. Patients were given OnabotulinumtoxinA based on the clinical needs and were consented to receive the treatment based on PREEMPT study protocol (Blumenfield et al. 2010). Patients with analgesic overuse were offered OnabotulinumtoxinA as recommended by the International Headache Society (Silberstein et al. 2008). There was no randomisation as the treatment was offered solely based on their clinical needs. Only patients with complete data were included for analysis. Patients were categorised into those with medication overuse (MO) and without overuse (WMO).

Medication overuse was defined according to the ICHD II. They were categorised as CM with medication overuse if:

- They had taken simple painkillers (paracetamol/non-steroidal anti-inflammatory drugs for ≥15 days.
- They had taken triptan, combination analgesics, opiates or in combination with simple painkillers for ≥10 days.

### Study design

**Fig. 1:**
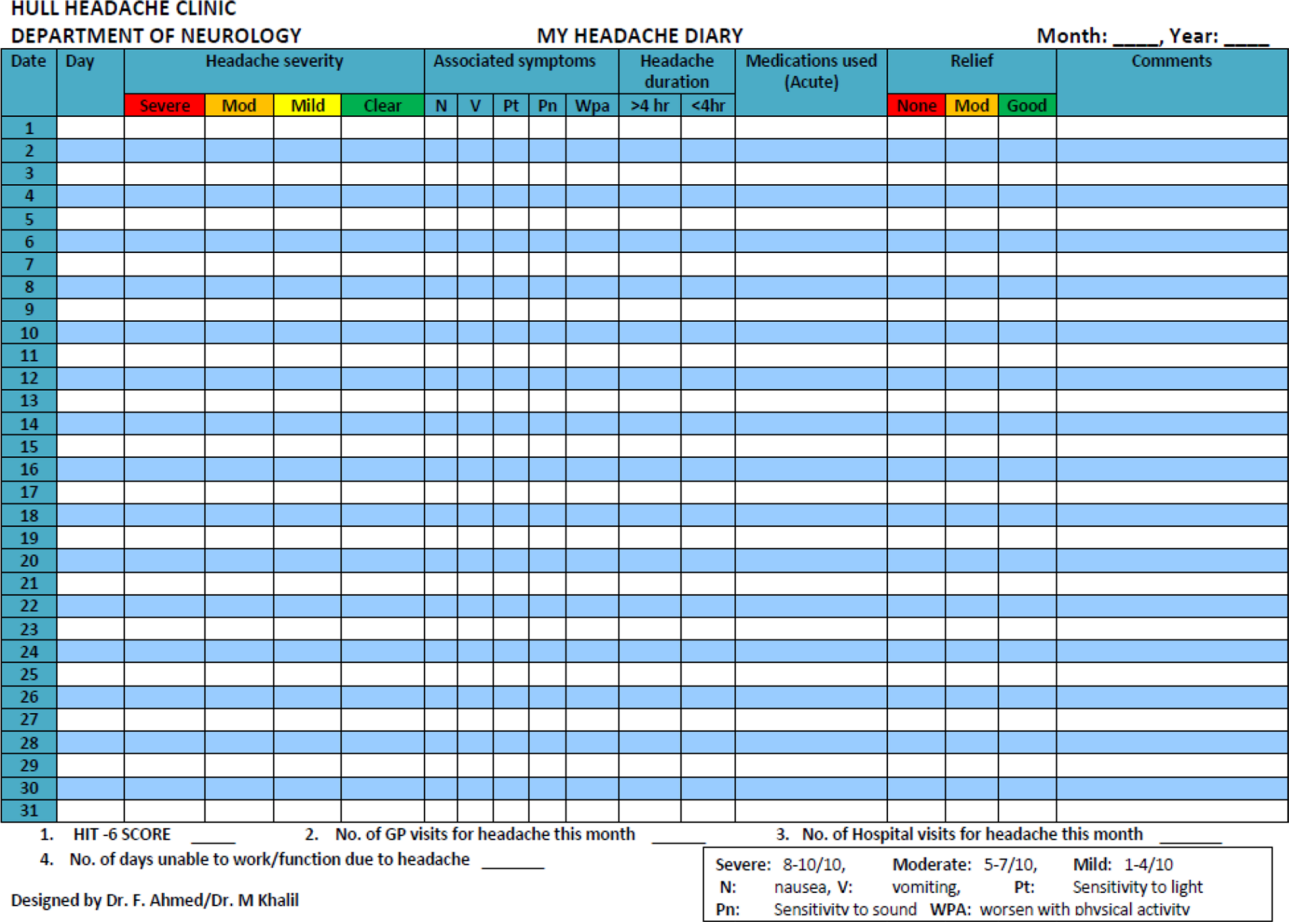
Hull Headache diary

Patients were injected OnabotulinumtoxinA according to the PREEMPT protocol, i.e., 155 units in 31 sites around the head and neck (Blumenfield et al. 2010). Although the PREEMPT paradigm allows up to further 45 units on the follow the pain sites, none of our patients received additional injections. Patients were asked to maintain a headache diary for at least 30 days prior to and continuously after receiving treatment. The Hull Headache Diary (shown below) (Fig. 1) was used to capture data on headache (Ahmed and Khalil 2013). The continuous diary filling was mandatory to assess response to treatment in order to determine whether patients were offered a repeat treatment. Patients who did not bring their diary or filled incompletely were asked to return with further 4 week of diary recording.

### Study measures

Assessments were made from completed diaries for headache days, migraine days, headache-free days; also of analgesic consumption for all painkillers before and after treatment. Quality of life was measured through the Headache Impact Test (HIT-6) on the day of treatment and 4 weekly afterwards. Responders were defined according NICE criteria i.e., at least 30 % reduction in headache days in the month following treatment. However, as some patients had reported improvement in migraine days alone, we evaluated the response based on Hull Criteria (Khalil et al. 2014) defined as:

- 50 % reduction on either headache or migraine days OR.
- Increments in headache free days twice that of the baseline in a 30 day period. Those with less than 3 days of headache free days were only classed as responder if they had achieved a minimum of six headache free days in the month after treatment.

50 and 75 % responder rates for each of the parameters (headache, migraine, headache-free days) were made and compared for each group.

### Statistical methods

Data was collected from patients undergoing their first full cycle of treatment. Patients were categorised into MO and WMO and the effect of treatment in each of these groups was examined separately, and a comparison of the two groups was also made.

The first analyses compared the differences in outcomes between measurements made before and after treatment for MO and WMO separately. All outcomes were measured on a continuous scale. An examination of the distribution of these outcomes found that they were skewed in their distribution at each set of measurements, and in terms of the change in values from pre to post treatment. As a result of these skewed distributions, the Wilcoxon matched-pairs test was used to compare the change in values over time. The analyses were first performed for all patients combined.

Secondly a comparison of the changes from pre to post treatment between MO and WMO was made. Due to the skewed distribution of the change values, Mann–Whitney test was used for the comparison.

For each patient it was calculated whether they were a ‘responder’ based on either a 50 % reduction in the number of days with symptoms, or a 75 % decrease. The exception was for headache free days where a responder was defined by either two-fold or three-fold increase in the number of headache free days. The Chi-square test was used to compare the proportion of responders between MO and NMO.

Hit-6 was used to quantify the change in QoL. The HIT-6 score was analysed on a continuous scale, and an examination of the change in values over time indicated that the changes were normally distributed. As a result the paired t test was used to compare the HIT-6 values before and after treatment for each group. To compare the outcome in the two groups the unpaired t test was used due to the normal distribution of the change values.

## Results

### Demographics and baseline headache characteristics

Of a series of 465 patients, full data on analgesic use was available on 434 patients (76 male, mean age 47.5; range 19–77, 358 females, mean age 44.9; range 18–91). Patients had a diagnosis of CM for a mean of 7.4 years (range 0.5–67). 219 (50.34 %) patients were overusing painkillers (MO). The demographics of the two groups are given in Table 1.

**Table 1:** Demographic details of patients with or without medication overuse

	All patients (N = 434)	MO (N = 219)	WMO (N = 215)
Female (N) Age	358 44.9 (18–91)	187 45.8 (18–91)	171 44 (18–77)
Male (N) Age	76 47.5 (19–77)	32 49.7 (18–74)	44 45.8 (23–77)
Age of onset of migraine	17	17	19
Duration of CM (years)	7.4 (0.5–67)	6	8

### Efficacy

Table 2 shows treatment outcome measures in CM patients without medication overuse (WMO). The results suggest statistically significant differences between the before and after treatment measurements for all outcomes examined.

**Table 2:** Outcome from before and after treatment for patients without medication overuse

Outcome	Patients (N)	Before treatment Median (IQR)	After treatment Median (IQR)	Change Median (95 % CI)	P value
Headache days	215	26 (20, 30)	17 (11, 28)	−5 (−7, −4)	<0.001
Migraine days	215	14 (10, 20)	8 (4, 12)	−6 (−6, −4)	<0.001
Crystal clear days	215	4 (0, 10)	13 (3, 19)	5 (4, 7)	<0.001
Painkiller days	215	8 (2,10)	4 (0, 8)	−1 (−2, 0)	<0.001
Triptan days	215	2 (0, 5)	0 (0, 4)	0 (0, 0)	<0.001
Days off work	43	3 (3, 5)	1 (0, 3)	−2 (−3, −1)	<0.001

Table 3 shows treatment outcome measures in CM patients with medication overuse (MO). The results suggest statistically significant differences between the before and after treatment measurements for all outcomes examined.

**Table 3:** Outcome from before and after treatment for patients with medication overuse

Outcome	Patients (N)	Before treatment Median (IQR)	After treatment Median (IQR)	Change Median (95 % CI)	P value
Headache days	219	28 (24, 30)	20 (12, 26)	−7 (−8, −5)	<0.001
Migraine days	219	16 (12, 20)	9 (5, 15)	−6 (−7, −5)	<0.001
Crystal clear days	219	2 (0, 6)	10 (4, 18)	7 (5, 8)	<0.001
Painkiller days	219	20 (16, 28)	10 (5, 18)	−8 (−9, −6)	<0.001
Triptan days	219	6 (0, 12)	2 (0, 7)	0 (−1, 0)	<0.001
Days off work	14	4 (2, 8)	2 (0, 4)	−2 (−5, 0)	0.04

The next analyses compared the change in outcomes from pre to post treatment between MO and WMO. The analysis results are summarised in Table 4, where the figures are the median (confidence interval) change for each group, along with P values indicating the significance of the results.

**Table 4:** Comparison of the treatment outcome measures in CM patients with or without medication overuse

Outcome	WMO		MO		P value
	Patients (N)	Median (95 % CI)	Patients (N)	Median (95 % CI)	
Headache days	215	−5 (−7, −4)	219	−7 (−8, −5)	0.15
Migraine days	215	−6 (−6, −4)	219	−6 (−7, −5)	0.58
Crystal clear days	215	5 (4, 7)	219	7 (5, 8)	0.15
Painkiller days	215	−1 (−2, 0)	219	−8 (−9, −6)	<0.001
Triptan days	215	0 (0, 0)	219	0 (−1, 0)	<0.001
Days off work	43	−2 (−3, −1)	14	−2 (−5, 0)	0.95

**Fig. 2:**
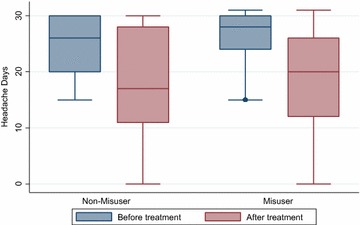
Headache days before and after treatment

**Fig. 3:**
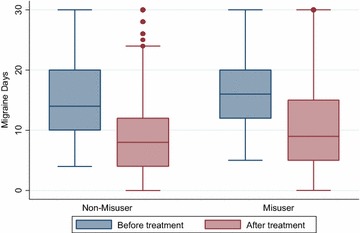
Migraine days before and after treatment

**Fig. 4:**
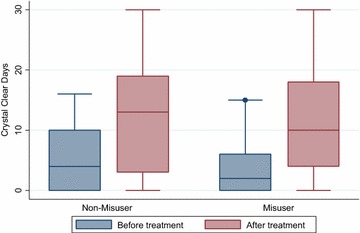
Crystal clear (headache free days) before and after treatment

The results suggest no difference in reduction of headache or migraine days in the two groups. The changes in crystal clear (headache free days) were similar. Patients with medication overuse showed significant reduction in consumption of analgesics after treatment for both triptans and simple painkillers. However, these results should be set in the context of different pre-treatment values for painkiller days, with higher pre-treatment values for misusers than for non-misusers. Figures 2, 3, 4 illustrate the outcome on headache, migraine and headache free days in the two groups before and after treatment.

**Fig. 5:**
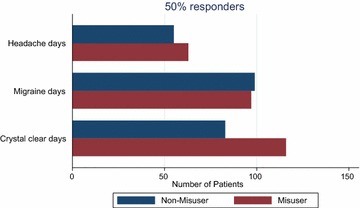
50 % response on three key outcome parameters

**Fig. 6:**
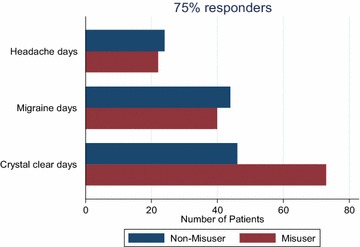
75 % response on three key outcome parameters

Table 5 shows a comparison of 50 and 75 % responder rate between the two groups. The results show no difference in reduction of headache and migraine days between the two groups. However, patients with medication overuse had more crystal clear days post-treatment than those without medication overuse. This may be attributed to fewer crystal clear days before treatment in those with medication overuse. Figures 5 and 6 graphically demonstrate the 50 and 75 % response rate in the two groups.

**Table 5:** 50 and 75 % responder rates comparison between MO and NMO patients

Outcome	Change	WMO, N (%)	MO, N (%)	P value
Headache days	≥50 % reduction	55/215 (26 %)	63/219 (29 %)	0.46
	≥75 % reduction	24/215 (11 %)	22/219 (10 %)	0.71
Migraine days	≥50 % reduction	99/215 (46 %)	97/219 (44 %)	0.71
	≥75 % reduction	14/215 (20 %)	40/219 (18 %)	0.56
Painkiller days	≥50 % reduction	64/215 (30 %)	94/219 (43 %)	0.004
	≥75 % reduction	33/215 (15 %)	44/219 (20 %)	0.20
Triptan days	≥50 % reduction	51/215 (24 %)	67/219 (31 %)	0.11
	≥75 % reduction	26/215 (12 %)	32/219 (15 %)	0.44
Days off work	≥50 % reduction	28/43 (65 %)	7/14 (50 %)	0.31
	≥75 % reduction	19/43 (44 %)	4/14 (29 %)	0.30
Crystal clear days	≥2-fold increase	83/215 (39 %)	116/219 (54 %)	0.003
	≥3-fold increase	46/215 (21 %)	73/219 (33 %)	0.005

## Quality of life

**Fig. 7:**
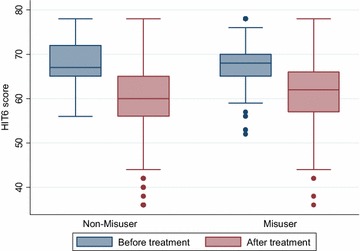
A graphical illustration of the pre and post treatment HIT-6 scores in the two sub-groups (MO and NMO)

Full HIT-6 score was available on 360/434 (82.9 %) patients (178 with medication overuse and 182 without medication overuse). The results suggested statistically significant differences between the before and after treatment scores in patients with or without medication overuse (see Tables 6, 7). A comparison of the changes from pre to post treatment between the two groups is shown in Table 8 (also see Fig. 7). The results suggested no strong evidence of a significant difference between the two sub-groups for the change in the HIT-6 score. However, there was weak evidence that the reduction was greater in patients without medication overuse, although this result was not quite statistically significant.

**Table 6:** HIT-6 scores before and after treatment in patients without medication overuse

Outcome	Patients (N)	Before treatment Mean (SD)	After treatment Mean (SD)	Change Mean (95 % CI)	P value
HIT6 score	182	68.1 (4.8)	59.7 (8.3)	−8.4 (−9.7, −7.1)	<0.001

**Table 7:** HIT-6 scores before and after treatment in patients with medication overuse

Outcome	Patients (N)	Before treatment Mean (SD)	After treatment Mean (SD)	Change Mean (95 % CI)	P value
HIT6 score	178	68.2 (5.1)	61.4 (17.8)	−6.8 (−8.0, −5.6)	<0.001

**Table 8:** A comparison of HIT-6 score before and after treatment between MO and NMO

Outcome	WMO		MO		P value
	Patients (N)	Mean (95 % CI)	Patients (N)	Mean (95 % CI)	
HIT6 score	182	−8.4 (−9.7, −7.1)	178	−6.8 (−8.0, −5.6)	0.08

## Discussion

Our prospective study provides data from patients treated with OnabotulinumtoxinA in a real-life setting at a tertiary headache centre in the United Kingdom since the publication of the PREEMPT study. The efficacy and safety data on 254 patients with an update on 465 patients have been presented recently (Khalil et al. 2014, 2015). This study reports the outcome on a large cohort stratified into those with and without medication overuse. However, our cohort in some ways was considerably different to the PREEMPT. All patients in our group had failed three preventive treatments based on the NICE recommendation for receiving OnabotulinumtoxinA (only 35 % in PREEMPT); baseline headache days were considerably higher in our population (27 versus 19.9 in PREEMPT) suggesting a more severely affected cohort in our study. However only 50 % patients in our study fulfilled the criteria for medication overuse (67 % in PREEMPT).

The prospective data in this study has shown no significant difference in the response to OnabotulinumtoxinA in patients with CM irrespective of analgesic consumption. The results show reduction in headache and migraine days before and after treatment in the two groups and increment in headache-free (crystal clear) days to be similar. The reduction in consumption of pain-killers was more in those with medication-overuse as there were more painkiller days before treatment in this group. There was no significant difference in 50 and 75 % responder rates in the two groups suggesting response to treatment is independent to baseline analgesic consumption. However, a two-fold and three-fold increase in headache-free days was more in the medication overuse group even though patients with medication overuse had less baseline headache days (26) and migraine days (14) than those without medication overuse (28 and 16 respectively). The number of headache-free days was also higher in those with medication overuse (4) than those without (2). This may be attributed to a direct effect on OnabotulinumtoxinA on analgesic consumption. Sandrini et al. (2011) have shown similar response in their cohort of 68 patients in a multi-centre double-blind, randomised, placebo-controlled, parallel group study (Sandrini et al. 2011). An increased sensitisation in pain processing (Perrotta et al. 2010) has been described in patients with medication overuse, and OnabotulinumtoxinA through inhibition of peripheral sensitisation (Aoki 2005) may influence central mechanisms responsible for facilitation in pain processing (Sandrini et al. 2011).

The results from this study are also consistent with the double-blind, randomised-controlled trial on topiramate in patients with CM, where reduction in migraine days from baseline were similar irrespective of analgesic consumption (Diener et al. 2007) and the PREEMPT sub-group analysis (Silberstein et al. 2013). This challenges the previous notion that preventive therapies are less effective in patients with medication overuse (Mathew 1993; Deiner and Limmroth 2004; Bigal et al. 2004; Katsavara et al. 2005).

Our data is prospective and open-label; the treatment was given based on the patients’ need and no blinding was done. A high placebo-response particularly with injectable treatments has been described (Diener et al. 2008) although this would have affected both groups and not influenced the overall results. In the same way improvement due to analgesic withdrawal would have been seen purely in the medication overuse group. Our patients represent what is seen in an average tertiary headache centre; the findings can, therefore, be projected to what clinicians see in other centres. We identified medication overuse based on the diary and identified painkillers as triptan or non-triptan. We, therefore, do not have data on consumption of opiates or other combination analgesics in our cohort.

## Conclusions

Preventive treatment is effective in patients with CM with or without medication overuse. OnabotulinumtoxinA considerably reduces the headache and migraine days whilst increasing headache-free days and the benefit is equally seen in those with or without co-existing medication overuse. We acknowledge the value of analgesic withdrawal although we suggest that this can be done alongside preventive treatment.
